# Revascularization Modalities in Acute Coronary Syndrome: A Review of the Current State of Evidence

**DOI:** 10.7759/cureus.47207

**Published:** 2023-10-17

**Authors:** Tahoora A Surve, Maitha A Kazim, Mehak Sughra, Agha Muhammad Wali Mirza, Siva Kumar Murugan, Karima A M Shebani, Fnu Karishma, Ishani Jayantibhai Trada, Mohammad Mansour, Kainat Asif, Loveneet Kaur, Amer Kamal, Nkechinyere Unachukwu, Aiman Naveed

**Affiliations:** 1 Internal Medicine, K. J. Somaiya Medical College, Mumbai, IND; 2 Internal Medicine, Rashid Hospital, Dubai, ARE; 3 Internal Medicine, Gujranwala Teaching Hospital, Gujranwala, PAK; 4 Internal Medicine, Jinnah Medical and Dental College, Karachi, PAK; 5 Internal Medicine, Meenakshi Medical College and Research Institute, Chennai, IND; 6 Internal Medicine, University of Zawia, Zawaiya, LBY; 7 Internal Medicine, Ghulam Muhammad Mahar Medical College, Khairpur, PAK; 8 Internal Medicine, American University of Barbados, Bridgetown, BRB; 9 General Medicine, University of Debrecen, Debrecen, HUN; 10 General Medicine, Jordan University Hospital, Amman, JOR; 11 Medicine and Surgery, Dr. Ruth K. M. Pfau Civil Hospital Karachi, Karachi, PAK; 12 Medicine and Surgery, Government Medical College, Patiala, IND; 13 Medicine, School of Medicine, The University of Jordan, Amman, JOR; 14 Medicine, East Tennessee State University, Johnson City, USA; 15 Internal Medicine, Mayo Hospital, Lahore, PAK

**Keywords:** immediate versus staged coronary revascularization, hybrid coronary revascularization, multivessel pci, culprit only pci, fractional flow reserve (ffr), intravenous ultrasound (ivus), : acute coronary syndrome, pci (percutaneous coronary intervention), coronary artery bypass grafting(cabg)

## Abstract

Acute coronary syndrome (ACS) stands as a leading global cause of mortality, underscoring the importance of effective prevention, early diagnosis, and timely intervention. While medications offer benefits to many patients, revascularization procedures such as coronary artery bypass grafting (CABG), percutaneous coronary intervention (PCI), and emerging hybrid approaches remain pivotal for ACS management. This review delves into the 2018 ESC/EACTS guidelines alongside an analysis of existing literature to shed light on the spectrum of revascularization methods. While both CABG and PCI demonstrate promising outcomes, the optimal choice between the two hinges on a comprehensive assessment of individual patient factors, anatomical complexity guided by advanced imaging, comorbidities, and age. The determination of whether to pursue culprit or total revascularization, as well as immediate or staged revascularization, is contingent upon various factors, including age, disease complexity, and clinical outcomes. This evidence-based decision-making process is orchestrated by a multidisciplinary heart team grounded in ongoing clinical evaluation. The primary objective of this review is to provide valuable insights into revascularization strategies and scrutinize the congruence of current guidelines with recent advancements in the field.

## Introduction and background

Acute coronary syndrome (ACS) refers to a range of conditions that result from an abrupt reduction in blood flow that results from partial or complete thrombosis of a coronary artery. It includes unstable angina, non-ST elevation myocardial infarction (NSTEMI), and ST elevation myocardial infarction (STEMI) [1].

Over seven million individuals worldwide receive an annual ACS diagnosis, marking a significant health concern [2]. As outlined by the WHO, cardiovascular diseases are the leading cause of death globally, taking an estimated 17.9 million lives each year [3]. Beyond fatalities, patients face an extensive range of complications and morbidities affecting multiple organ systems, showcasing the extensive impact on general health and the quality of life [4]. Therefore, to alleviate the strain on healthcare systems, a collective effort of prevention, early diagnosis, and prompt treatment is imperative.

The occurrence of ACS is attributed to a diverse array of factors, spanning both modifiable and non-modifiable risk elements. Both age-related physiological changes and accumulated cardiovascular risk factors predispose to ACS over time [5]. When compared to the younger population, ACS has poorer consequences for older adults [6]. Through the SCORE II algorithm, we gain insights into a patient’s 10-year cardiovascular event risk, with scores of ≥10% indicating a very high risk and scores of ≤1% signifying lower risk categories for ACS development [7]. Utilizing risk scores like the thrombolysis in myocardial infarction (TIMI) score and the global registry of acute coronary events (GRACE) can also provide valuable insights [8].

While pharmacotherapy has demonstrated its effectiveness in treating a majority of patients, the role of revascularization strategies remains crucial in ACS management. The two primary revascularization methods are percutaneous coronary intervention (PCI) and coronary artery bypass grafting (CABG). However, in line with recent guidelines, the hybrid coronary revascularization (HCR) approach, which integrates both procedures, is garnering attention. This innovative technique addresses multi-vessel coronary artery disease and is steadily gaining popularity [9].

Through this literature review, our intention is to furnish a comprehensive panorama of revascularization modalities applicable to ACS patients. We endeavor to equip surgeons with up-to-date insights in this domain. We have identified the indications of revascularization as determined by the ESC/EACTS guidelines [7]. We’ve additionally used the SYNTAX score II [10] to assess the anatomical complexity of the lesion and its role in the choice of the appropriate revascularization procedure. 

Recent advancements in coronary artery imaging modalities like intravascular ultrasound (IVUS) and fractional flow reserve (FFR) have provided new insights into the extent of coronary artery disease and the physiology of blood flow across the diseased vessels [11]. This review sheds light on the impact of IVUS and FFR on guiding revascularization strategies and outcomes.

Additionally, the optimal approach to revascularization in ACS remains debated. This review provides insight into whether culprit-only or complete revascularization is superior for patients with multivessel disease (MVD) in the setting of ACS [12,13] and review the evidence behind conventional immediate PCI vs. initially medically stabilizing ACS patients before staged revascularization [14,15]. Emphasis is placed on the impact of patient factors like age and the extent of disease in determining the risk-benefit ratio for each approach.

## Review

Revascularization modalities in acute coronary syndrome (ACS)

Revascularization is indicated in patients who receive medical treatment as per the ESC/EACTS guidelines [7] but still have persistent symptoms and in patients in whom there is a possibility of improvement in prognosis. Several studies prove that revascularization is more effective in improving symptoms of ACS and enhancing quality of life as compared to medical therapy alone [16]. This section sheds light on various revascularization modalities.

Coronary artery bypass grafting (CABG)

CABG remains a fundamental therapy in the treatment of coronary artery disease (CAD) due to its ability to provide lasting relief from symptoms and reduce the risk of adverse cardiac events. It utilizes vascular grafts to bypass stenotic coronary artery segments and restore myocardial perfusion.

Types of grafts 

The two main types of commonly used autologous arterial and venous grafts are left internal mammary artery (LIMA) and saphenous vein (SV) grafts, respectively [17]. LIMA is typically grafted directly from its origin to the coronary artery beyond the blockage, whereas the SV is anastomosed proximally to the aorta and distally to a coronary artery downstream from the blockage. The venous grafts are inverted to allow unobstructed blood flow through the venous valves.

Even though the average diameter of LIMA is smaller than SV, it has the benefit of retaining its patency and consequently improving survival [18]. It is also reported to have growth potential, which is evident from the greater diameter of old LIMA grafts [19].

The SV, on the other hand, has the disadvantage of declining patency with time and resulting in worse clinical outcomes such as recurrent angina, MI, repeated revascularizations, etc. [20]. According to a study, 13.6% of SV grafts were completely occluded after one year [18]. The factors that contribute to the long-term patency of saphenous grafts are grafting into LAD or in a vessel of 2mm in diameter [21]. The saphenous grafts are still widely used owing to their benefit of being larger in size and, therefore, being easy to handle [22]. Moreover, they have lower transfusion requirements compared to bilateral internal mammary artery grafts [23].

On-pump vs. off-pump coronary artery bypass grafting

On-pump CABG is the traditional method of performing surgery where a cardiopulmonary bypass allows surgery to be performed in a bloodless and motionless field that allows precise graft placement and grafting of poorly accessible fields. In contrast, off-pump CABG is a relatively newer procedure that uses mechanical stabilizers and special instruments to give a still field for surgery. However, it provides limited field access and a relatively unstable field, which makes graft placement challenging [24]. On-pump CABG allows comprehensive revascularization of multiple arteries in a single procedure, whereas off-pump CABG requires multiple surgeries [25]. The principal drawback of off-pump CABG is the potential need to institute cardiopulmonary bypass due to inadequate cardiac exposure, the expectation of technically challenging coronary anastomoses, or hemodynamic instability during regional myocardial ischemia [26].

In terms of safety, off-pump CABG outpaces on-pump CABG, as it is associated with a decreased systemic inflammatory response and a low risk of bleeding and stroke. It also reduces the risk of short-term mortality [27]. However, its long-term mortality benefit is questionable. A pooled analysis of 22 studies shows a 7% rise in long-term mortality with off-pump CABG (hazard ratio (HR) 1.07; 95% confidence interval (CI), 1.03-1.11; P=.0003) [28].

Minimally invasive coronary artery bypass grafting

The drawbacks of open CABG in terms of prolonged recovery time, unsightly scarring, chronic pain, and potential sternal and wound complications [29] underscored the need for minimally invasive techniques [30]. They involve small incisions that avoid sternotomies, allow better precision, and improve the surgical experience.

Recent developments include the endoscopic CABG, where the endoscope is utilized for harvesting and placement of the graft. A study evaluating endo-CABG indicates a success rate of 97% with minimal MACE as low as 5% at six-month follow-up [31]. Robotic surgery is also successful in performing precise grafting through small incisions.

These procedures are safe and feasible [32] and have the advantages of a low risk of infection, a short hospital stay, and better clinical outcomes [33]. They are suitable for patients with risk factors such as diabetes, morbid obesity, thoracic deformity, and the elderly [33]. However, they are technically very challenging and uneconomical [34]. Although no explicit patient selection criteria exist for these procedures, the choice of approach depends on patient preference, surgeon experience, and available resources.

In cardiovascular medicine, CABG has undoubtedly shaped the landscape of cardiac care, demonstrated low mortality rates and improved long-term survival. It remains the optimal approach for patients with complex CAD [35]. Further advances balancing efficacy, safety, and cost will shape the future evolution of CABG as a transformative and dynamic field.

Percutaneous coronary intervention (PCI)

Percutaneous coronary intervention utilizes a minimally invasive approach in which a catheter introduced through a peripheral artery is navigated to the stenosed blood vessel. Specialized techniques are then utilized to open up the narrowed artery and restore its blood flow. It is commonly performed to relieve chest pain symptoms in patients with ACS and to prevent heart attacks. It has revolutionized the treatment of CAD and is now a standard procedure in interventional cardiology [36].

Balloon angioplasty

The first successful balloon angioplasty was performed in 1977 by Dr. Andreas Grüntzig [37]. This technique has been a significant advancement in the treatment of CAD and has played a crucial role in the success of PCI procedures. It involves the insertion of a deflated balloon through a peripheral artery, which is then inflated in the narrowed segment of the vessel, widening its lumen and relieving the obstruction to blood flow by disrupting the atherosclerotic plaque [37].

The procedure had its drawbacks, i.e., the risk of restenosis [38]. Therefore, it is no longer the primary intervention for the management of ACS. However, it is still performed in cases where stent placement is not feasible or can lead to harmful consequences [39,40]. Additionally, it has paved the way for CABG and future PCI procedures [36].

Drug-coated balloons

The risk of restenosis with balloon angioplasty drove the creation of drug-coated balloons (DCBs). The balloon is loaded with anti-proliferative drugs such as paclitaxel and sirolimus [41]. By delivering the drug directly to the artery wall during balloon inflation, high tissue concentrations can be achieved at the site of injury while minimizing systemic exposure [42]. In the management of in-stent restenosis and small vessel < 3mm disease, the effectiveness of DCBs is comparable to drug-eluting stents (DES) [43]. However, the role of DCBs in treating large vessel disease and bifurcation lesions requires more evidence.

Clinical trials show that DCBs reduce the rates of target lesion revascularization and major adverse cardiac events (MACE) as compared to balloon angioplasty alone. In patients with small vessel disease, the BELLO trial [44] indicates that PCBs (paclitaxel coated balloon) have reduced late lumen loss (0.08 ± 0.38 mm vs. 0.29 ± 0.44 mm) and the risk of MACE (10% vs. 16.3%; P = 0.21) at 12 months compared to PES (paclitaxel eluting stent) [44].

Similarly, in patients with in-stent restenosis, a trial [45] proved the non-inferiority of DCBs, as the risk of segment late lumen loss (0.38 ± 0.61 mm vs. 0.17±0.42 mm; P = 0.03) and restenosis (20% vs. 7%) was observed to be greater in the PES group as compared to the PCB [45]. The rate of MACE was also greater in PES (22% vs. 9%, P = 0.08) at 12 months [45].

DCBs have the additional benefit of no stent-related complications, as with stent therapy. Additionally, it requires a shorter duration of dual antiplatelet therapy (DAPT) in contrast to DES, proving its usefulness in patients with higher bleeding risks [46].

Laser angioplasty

Laser angioplasty employs a catheter that emits high-energy radiation to vaporize the atherosclerotic plaque, occluding the diseased vessel [47]. This technology has made progress since its inception in the 1960s [47]. The excimer laser is associated with better outcomes as compared to the conventional Nd:Yag and argon lasers. The development of laser-tipped guidewires further improved the outcomes of laser angioplasty [47]. A retrospective study at a tertiary care center signifies that laser angioplasty had a high success rate of 81.6% and a low complication rate, as evident by only 0.6% of coronary vessel perforations attributed to laser angioplasty [48]. Nonetheless, laser angioplasty never became a dominant method over traditional balloon angioplasty and stenting due to its technical complexity, long procedure time, and restenosis rates.

Stenting

This commonly used procedure involves the insertion of expandable mesh tubes called stents in the blocked vessels. Initially, bare metal stents were utilized for stenting, but they were associated with a high risk of stent thrombosis and required prolonged DAPT. These problems facilitated the inception of DES, where anti-proliferative drugs are incorporated in the stents, effectively mitigating the risk of stent thrombosis and restenosis [49]. Drugs like Everolimus, Sirolimus, Zotarolimus, Biolimus, and Paclitaxel are commonly used [50,51].

Multiple studies suggest that DES demonstrate superior efficacy to bare metal stents, as shown by a trial where the rate of revascularization was observed to be lower with DES (16.5%) as compared to bare metal stents (19.8%) with an absolute risk reduction of 3.3% with DES (HR 0.76; 95% CI, 0.69-0.85; P<0.001) [52]. Similarly, the common complication of stent thrombosis was also observed to be lower in DES (0.8% vs. 1.2%, P=0.0498). The combined rate of mortality and nonfatal MI was higher in bare metal stents at 17.1% when compared to DES at 16.6% (HR 0.98; 95% CI, 0.88-1.09; P=0.66) [52]. However, this difference was statistically non-significant.

Thrombectomy

In this procedure, aspiration catheters and mechanical techniques are used to remove the thrombus occluding the vessel [53]. It is preferred for treating acute MI or STEMI [53]. It reduces myocardial muscle damage and improves clinical outcomes.

Role of atherectomy in percutaneous coronary intervention

Atherectomy involves the removal of atherosclerotic plaque from the diseased vessels. It is primarily employed for dense, heavily calcified lesions that are not successfully managed with angioplasty or stenting alone [54]. It enhances the success rate of PCI by optimizing stent placement. Multiple techniques are used to remove or destroy the plaque. One of those is rotablation (RA), where a high-speed rotating burr is used to remove the calcified plaque [55].

In patients with heavily calcified coronary lesions who had undergone RA-assisted PCI [56], a retrospective analysis demonstrated a success rate of 99.4% and good short- to intermediate-term results [56]. However, there was a relatively high rate of periprocedural complications. Similarly, another study showed a success rate of 96.4% of RA-assisted PCI with in-hospital MACCE of 10.8%. at 1.5-year follow-up [57].

Incorporating rotational atherectomy into PCI procedures may improve outcomes for patients with calcified lesions and enhance the overall prognosis. Ongoing studies assessing outcomes, indications, and procedural guidance will help clarify the appropriate role of atherectomy and determine the appropriate criteria for its use.

Imaging guide for percutaneous coronary intervention

The role of medical imaging has taken center stage, revolutionizing the way we approach complex procedures like PCI. These modalities have emerged as indispensable companions, which, when expertly wielded, serve as the guiding lights illuminating the intricate pathways of coronary vessels and profoundly influencing the decisions made during the revascularization procedures [58].

The technologies range from angiography and intravascular ultrasound (IVUS), which offer an unparalleled view inside the arteries, to fractional flow reserve (FFR), which quantifies the functional significance of stenosis. Other imaging techniques, like intracardiac echo (ICE), which enables real-time images of the heart, near-infrared spectroscopy (NIRS), which assesses plaque composition, and optical coherence tomography (OCT), which unveils microscopic details of vessel walls, are also gaining popularity [58,59]. These techniques aid in charting the most appropriate course for revascularization. 

Intravenous ultrasound vs. fractional flow reserve

An interesting recent development that requires attention is the FFR and IVUS. FFR offers functional data indicating whether a lesion is hemodynamically significant and gives a quantitative effect of stenosis on coronary blood flow, whereas IVUS offers anatomical details of the lesion, including its size and composition [60]. FFR helps guide decisions on whether to perform PCI, and IVUS assists in stent selection and optimization during PCI. FFR is typically measured during the procedure, while IVUS can be used before and during the procedure [61].

An RCT by Koo et al. [60] showed the non-inferiority of FFR-guided PCI, as the frequency of PCI was lower with FFR at 44.4% as compared to IVUS at 65.3%. Moreover, the risk of mortality, MI, and revascularization and patient-reported outcomes were noted to be similar between the two groups. These results reflect the promising potential of FFR-guided PCI [60].

A meta-analysis comparing IVUS vs. other imaging techniques revealed a tendency in favor of IVUS over FFR in terms of reducing the risk of MACE (Odds Ratio (OR) 0.75; 95% CI, 0.52-0.88 vs. OR 0.81; 95% CI, 0.64-1.02; respectively), stent thrombosis, revascularization, and subsequent MI in ACS patients [62].

Although the two emerging technologies have promising outcomes in guiding PCI, the differences demonstrated by these studies raise the need for more evidence to firmly establish the respective roles of FFR and IVUS in contemporary PCI guidance and determine which patients may derive greater benefit from one modality over the other.

Hybrid coronary revascularization (HCR)

For many years, PCI and CABG have remained the primary treatments for CAD. However, the best revascularization method for the MVD remains a topic of controversy. In the mid-1990s, HCR emerged in the hopes of providing the “best of both worlds” [63]. The aim is to provide long-term survival while decreasing surgical trauma and adverse cardiovascular events. It involves the grafting of the LIMA to the left anterior descending (LAD) artery along with PCI to non-LAD vessels. Therefore, patients who have multiple vessel diseases (MVD) can benefit from these two combined processes. 

According to 2018 European Heart Journal guidelines [64], HCR was considered one of the most suitable revascularization strategies for MVD where proximal LAD or left main is eligible for surgical LIMA grafting and non-LAD vessels are suitable for PCI, in MVD that is not fully revascularized by PCI, or where LAD is not amenable to PCI while other non-LAD lesions are suitable for PCI.

HCR combines the beneficial outcomes of PCI and CABG while minimizing the associated risks owing to its less invasive approach, shorter duration of hospitalization, and fewer post-operative complications [65,66]. Additionally, HCR offers more complete revascularization than either procedure alone. The greatest benefit of HCR was noticed in patients with a less complicated coronary vasculature layout, as tortuous vessels could pose challenges for stent placement [65].

Although HCR has been available for over two decades now, it remains limited to specialized centers as it necessitates the collaboration of a heart team consisting of interventional cardiologists and cardiac surgeons to identify eligible patients and perform the procedure effectively. Patient selection for HCR depends on patient factors and disease factors, keeping in mind the risks and benefits for the patients. However, a definitive criteria for patient selection is still lacking [66].

Selection of revascularization strategy

As per the ECS/EACTS guidelines [7], revascularization is recommended for classes IA, IB, and IIB [67,68]. In order to select the revascularization modality, certain anatomical, clinical, and technical aspects should be considered [67]. The risk of surgical mortality is a significant concern, and scores such as the EuroSCORE II and STS were employed to assess this risk, but they also had certain limitations [69]. Later, the SYNTAX trial introduced the SYNTAX score, which predicted MACCE and death following PCI (not CABG) [70,71]. In 2020, the SYNTAX score II was introduced, providing insights into MACE (at five years) and 10-year mortality [10].

Role of anatomical complexity

In choosing the right revascularization therapy, coronary anatomical complexity is a key factor reflecting lesion severity [68]. It should be assessed because CABG is favored for severe calcification and in-stent restenosis, while PCI is preferable for extremely small distal coronary targets [68]. Notably, studies, including the original SYNTAX trial, have shown that PCI tends to have higher rates of MI (9.7% vs. 3.8%; P <0.0001) and repeat revascularization (25.9% vs. 13.7%) than CABG [35]. Notably, for patients who had low SYNTAX scores, PCI was comparable to CABG with little variation in patient outcomes except the high repeat revascularization rates with PCI (25.4% vs. 12.6%, respectively; P < 0.001) [71].

Left Main Stenosis

The decision on the suitable revascularization procedure for left main artery stenosis depends upon the SYNTAX score. CABG is favored over PCI for intermediate and high SYNTAX scores [35]. This is supported by the SYNTAX trial, which highlighted a greater risk of MACCE in PCI (36.9%) than in CABG (31.0%). This finding was particularly evident in patients with intermediate (36.0% in PCI vs. 25.8% in CABG; p=0.008) and high SYNTAX scores (44.0% in PCI vs. 26.8% in CABG; p<0.0001). These results reinforce the significance of choosing the best treatment approach [70]. These findings were further supported by the EXCEL patient characteristics and lesion complexity trial [72], whose results indicate that the risk of all-cause mortality was also higher with PCI than with CABG (16.3% vs. 10.9%; P=0.049) [72].

Left Anterior Descending Stenosis

For LAD stenosis with single and double-vessel disease, PCI is usually preferred over CABG. CABG is the optimal treatment approach for proximal LAD stenosis and one- or two-vessel disease, as both conditions fall into the same category according to ESC/EACTS guidelines [67]. According to the anatomical complexity data from the Kapoor trial [73] and a subsequent meta-analysis on patients with proximal LAD stenosis, the all-cause mortality rates at five years were similar between PCI and CABG (90.6% vs. 92.8%). However, the rate of repeat revascularization was significantly higher with PCI as compared to CABG (33.5% vs. 7.3%; 95% CI, 20.1-33.3) [72,73].

Multivessel Disease

Patients with lesions in more than two coronary arteries are considered to have multivessel disease (MVD). For those with MVD and a SYNTAX score >22, CABG is the recommended choice. However, if the SYNTAX score is ≤22, both revascularization strategies, PCI and CABG, can be considered [67,74]. According to a study, PCI was observed to be associated with low efficacy and safety in MVD as it had a higher risk of revascularization (25.4% vs. 12.6%; P < 0.001) and mortality (14.6% vs. 9.2%; P= 0.006) than CABG [71].

This is further evidenced by a meta-analysis where PCI demonstrated a decreased risk of all-cause mortality (RR 0.72; 95% CI, 0.64 - 0.81; P <0.001) when compared to CABG (RR 0.70; 95% CI, 0.61- 0.80; P <0.001). This suggests that PCI might offer a slight edge in terms of survival [75].

Role of Comorbid Conditions

Patients with contraindications for surgery may be better suited for PCI. A 10-year follow-up of the SYNTAX II trial revealed higher mortality rates among the elderly, females, and those with comorbid conditions, particularly in cases of complex CAD [10,74].

Furthermore, a six-year follow-up of the Chen trial reported a higher rate of MACCE in diabetics compared to non-diabetics who underwent PCI. All-cause mortality was also higher in diabetic patients. In contrast, a study demonstrated an increased risk of stroke in diabetics who were treated with CABG (2.2% vs. 0.6%; P = 0.003) [76]. Therefore, patients with diabetes are generally recommended to undergo CABG, but more evidence is required to validate this conclusion. Additionally, factors such as contraindications for DAPT and reduced left ventricular function tend to favor CABG over PCI [77]. 

Extent of Occlusion

Another factor that determines the choice of a revascularization procedure is the extent of occlusion. In the case of chronic total occlusion (CTO), a study by Zaman et al. [78] concluded that CABG results in a higher rate of complete revascularization (CR) than PCI (67% vs. 32%, P < 0.0001) [78].

*Financial Perspective* 

From a fiscal perspective, CABG may be a more financially suitable treatment for patients with MVD, while PCI may serve as a more economical option in cases of less complex disease. Index revascularization procedure costs per patient post-left main intervention were observed to be $4,850 greater with CABG. Similarly, total index hospitalization costs were $17,610 higher with CABG than PCI (P < 0.001). The trend was persistent for cumulative five-year costs. CABG has been predicted to raise the lifetime costs per patient by approximately $21,551 [79]. However, insurance coverage may play a role in modulating this aspect of the decision-making process. 

Percutaneous coronary intervention vs. coronary artery bypass grafting

While CABG may not be the preferred choice for LMCA disease, it remains the standard of care for MVD and complex lesions [71]. It may benefit diabetic patients with MVD, while PCI is suitable for single-vessel LAD stenosis. Mortality and event rates vary, emphasizing the need for personalized decisions based on patient characteristics and lesion complexity. SYNTAX score II supports CABG for three-vessel disease, with contraindications, comorbidities, and diabetes status factoring into the choice.

While there have been a host of studies conducted on the comparison between bare metal stents and CABG, fewer studies exploring the efficacy of DES in comparison to CABG are available. Given the numerous merits and demerits associated with each treatment, further evidence is required to determine the most appropriate intervention strategy for varying manifestations of CAD.

Hybrid coronary revascularization vs. percutaneous coronary intervention

In choosing between the two as a better revascularization modality, multiple trials and observational studies have been conducted. According to a study, HCR reduces the risk of repeat revascularization as compared to PCI (91.13% vs. 83.59%; aHR = 0.51; 95% CI, 0.34−0.77) [80]. However, the risk of mortality [81] and MACCE [82] were statistically non-significant across multiple trials and observational studies. An interesting finding was observed: the patients who underwent HCR were not given a P2Y12 upon discharge despite stent placement [81].

These findings were further substantiated by a meta-analysis of seven studies, which showed that the efficacy of HCR and PCI is comparable. The risk of revascularization was decreased with HCR (OR 0.49; 95% CI, 0.37-0.64; P < 0.001). Interestingly, the incidence of MI was also low with HCR (OR 0.40; 95% CI, 0.20-0.80; P = 0.010). However, the safety concern of HCR remained unsettled, as there was no significant difference observed for other MACCEs (OR 0.46; 95% CI, 0.20-1.05; P = 0.061) and long-term outcomes [66]. Due to the non-invasive nature of HCR, it has fewer post-operative complications of blood transfusion and infections than those observed with PCI.

Hybrid coronary revascularization vs. coronary artery bypass grafting

HCR can be a fine replacement for CABG for patients with MVD, as HCR has the advantage of a short hospital stay (weighted mean difference (WMD) -1.77 days; 95% CI, -3.07-0.46; P = 0.008) and an ICU stay of 20 hours less than CABG (WMD -17.47 hours; 95% CI, -31.01--3.93; p = 0.01) [65]. However, a meta-analysis presented a high risk of repeat revascularization in the short-term (OR 3.28; 95% CI, 1.62-6.64; P < 0.001) and mid-term (OR 2.84; 95% CI, 1.64-4.92; P < 0.001) follow-up [9] with HCR. It also showed a higher rate of mortality with CABG (OR 0.35; 95% CI, 0.18-0.69; P = 0.002) [83].

Another meta-analysis indicates that there is no statistically significant difference between HCR and CABG in the risk of MACCE during hospitalization (OR 0.68; 95% CI, 0.34-1.33). and the risks of MI, stroke, and mortality. These results are consistent across numerous trials and meta-analyses [65].

HCR has gained some popularity due to its effectiveness and being less invasive. While it offers a shorter hospital stay than CABG, it increases revascularization risk. Similarly, it reduces the risk of revascularization when compared with PCI, but safety remains a concern. Current evidence on HCR is limited, and more studies are required to refine its selection criteria and evaluate its long-term outcomes.

Culprit vessel vs. total revascularization

The emergency management of STEMI consists of immediate revascularization of the culprit vessels, but there are no dedicated prospective trials for non-ST-segment elevation ACS (NSTE-ACS). In MVD, managing non-culprit lesions (NCL) and selecting revascularization techniques are complicated when dealing with both ACS and chronic coronary syndrome (CCS). Adopting a complete revascularization (CR) strategy for MVD emerges as a medically feasible alternative, aiming to decrease ischemia, alleviate symptoms, and reduce cardiovascular risks. The debate of culprit only vs. CR is centered on finding a balance between minimizing procedural risks and maximizing long-term patient outcomes. Factors such as the patient’s age, mortality, morbidity benefits, and cost-effectiveness impact the choice of the procedure. 

An observational study [84] that pools data from RESCUE and SMC-ECMO registries shows a 30-day mortality benefit and a lower risk of renal replacement therapy with MV-PCI over CO-PCI (54.3% vs. 68.0%; P = 0.018). Studies are also indicating an increasing MV-PCI preference amongst physicians as part of a treatment strategy for NST-ACS, which may reduce the incidence of new indications for coronary revascularization during follow-up [85].

However, there are contrasting views on the two choices, as another study shows no superiority of MV-PCI over CO-PCI at one-year post-op follow-up in terms of safety and effectiveness with respect to the risk of mortality or MI (HR 0.90; 95% CI, 0.69-1.17) and repeat revascularization (HR 0.76; 95% CI, 0.55-1.05) [85]. Additionally, Brener et al. [86] declared that a patient’s operator prefers CO-PCI over MV-PCI (68% vs. 32%) due to higher short-term procedural success (91% vs. 88%; P < 0.001) [86]. This study had limitations, primarily related to the lack of long-term follow-up [86]. In terms of cost-effectiveness, the CULPRIT-SHOCK trial favors CO-PCI over MV-PCI [12].

Rationale for age: MVD has shown an increasing trend over the last decade, from 26% in 2005 to 36% in 2015. It is associated with the worst prognosis in advanced age. A detailed meta-analysis on non-ST elevation acute coronary syndrome (NSTEACS) reveals that MV-PCI was comparable to CO-PCI in terms of mortality (HR 0.79; 95% CI, 0.58-1.09) and the composite of death and MI (HR 0.90; 95% CI, 0.69-1.17) [86]. Surprisingly, the pharmacological approach also didn’t significantly benefit elderly patients in reducing CVD-related complications. This suggests the potential for a CR approach. 

In contrast, the DANAMI-3-PRIMULTI trial concludes that in patients <75 years, CR reduces the incidence of mortality, risk of reinfarction, and revascularization. Whereas in patients aged ≥75 years, there was no significant difference between the two revascularization strategies. Additionally, the benefit of CR attenuated with age (P=<0.001) [13].

Such results showing the declining impact of CR with age cannot be generalized as there is an underrepresentation of the elderly in such studies, as the mean age of patients is usually 64.1 ± 12.4 years [87]. Additionally, comorbid conditions in the elderly impact their baseline risk and outcomes. There is a higher procedural risk during revascularization, including bleeding, kidney injury, and periprocedural myocardial infarction. Therefore, the harm-versus-benefit balance for aggressive revascularization strategies may differ for the elderly. Moreover, the endpoint of care for the elderly is more focused on quality of life and symptom relief than on longevity. The priorities that guide revascularization choices may, therefore, differ in the elderly population [13,88,89].

The FIRE trial, which aims to bridge this research gap [88], targets the elderly population >75 years of age with STEMI or NSTEMI who have a MVD [85]. A recently published RCT concluded that MV-PCI was associated with a lower risk of mortality than CO-PCI [89]. Additionally, the study showed that the benefits of complete revascularization (MV-PCI) were consistent across different levels of vessel stenosis severity.

Although the AHA/ACC guidelines [85,90] recommend MV-PCI over CO-PCI due to the reduced likelihood of recurring cardiac events, all-cause mortality, and MACE, the debate still lacks evidence due to a lack of clinical trials and studies in this scope of interventional cardiology. Additional clinical trials across diverse age demographics are warranted to further comprehend age-related complexities in heart disease and refine therapeutic approaches. This will become increasingly relevant given shifting demographics and rising life expectancies stemming from advanced treatments. Well-designed, prospective studies can help clarify optimal standards of care for distinct patient age groups in the future.

Immediate vs. staged complete revascularization

Although robust data supports the benefits of complete revascularization in ACS, particularly STEMI, refining the ideal evaluation of NCL and the timing of CR remains an ongoing challenge [91]. Multiple RCTs showed far more rising benefits from staged or immediate CR than culprit-only PCI [92].

Immediate complete revascularization refers to revascularizing all blocked coronary arteries at the initial hospitalization. This approach is adopted when there is a significant blockage in multiple coronary arteries, hemodynamic instability, and high-risk symptoms such as ongoing chest pain and signs of myocardial damage [93].

Staged complete revascularization, on the other hand, refers to the initial revascularization of the culprit vessel and subsequent revascularization of the non-culprit vessels during the hospitalization or over the next six weeks [15]. Therefore, it is preferred in elderly patients with comorbidities a single critical culprit lesion ACS or when there are limited resources and scheduling constraints [94]. 

In the BIOVASC trial by Diletti et al. [14], the rate of mortality, MI, and ischemia-driven revascularization was greater with immediate complete revascularization than staged complete revascularization (7.6% vs. 9.4%) at one-year follow-up. These results imply that immediate, complete revascularization could potentially be an excellent alternative to staged revascularization [14].

As far as safety is concerned, a trial ascertained one-year outcomes comparing immediate and staged complete revascularization [95]. Even though the mortality rates in the two groups were noticeably different (albeit statistically non-significant), favoring the staged complete revascularization group (9.7% vs. 2.8%; HR 3.53; 95% CI, 0.97-12.84), the incidence of MACE exhibited no statistically significant difference between the two [95]. The trial was halted early and did not yield a conclusion, presumably due to limited power [95].

To sum up, the complete revascularization approach has been proven to have a better prognosis than the culprit-only PCI approach. However, to ascertain the optimal timing of complete revascularization (immediate vs. staged), more studies are required, as there are a limited number of studies with inconclusive data and a limited number of patients [96].

## Conclusions

PCI and CABG have been the standard revascularization techniques for treating CAD. While both techniques are still evolving and adapting more effective strategies to improve outcomes, the hybrid revascularization technique has shown promising results in treating high-risk CAD patients. More extensive studies are warranted to answer questions about the use and indications of these techniques. Additionally, various imaging modalities have been in use that guide revascularization techniques. Among these, the FFR and IVUS have been heavily compared. They have demonstrated promising outcomes in guiding PCI, but the choice of one over the other is not well established yet and warrants more studies to be conducted. Furthermore, CR has shown a better overall outcome in comparison to CO-PCI. Nonetheless, studies have demonstrated that the benefit of CR decreased with age, but the age group selected in most trials isn’t completely representative of the elderly population. In terms of choosing immediate or staged CR, neither has demonstrated superiority in the recent trials. Nonetheless, the optimal timing of CR warrants more clinical evidence, as the existing data is inconclusive.
